# The relationship of social support concept and repeat mammography among Iranian women

**DOI:** 10.1186/s12905-015-0253-7

**Published:** 2015-10-24

**Authors:** Fariba Farhadifar, Parvaneh Taymoori, Mitra Bahrami, Shamsy Zarea

**Affiliations:** Social Determinants of Health Research Center, Kurdistan University of Medical Sciences, Sanandaj, Iran; Social Determinants of Health Research Center, Kurdistan University of Medical Sciences, PO Box 66177–13391, Pasdaran Street, Sanandaj, Iran; Department of Genicology, School of Medicine, Kurdistan University of Medical Sciences, Sanandaj, Iran

**Keywords:** Breast cancer, Repeat mammography, Social support, Iranian women

## Abstract

**Background:**

Breast cancer ranks as the first most common cancer among the Iranian women. The regular repeat of mammography with 1–2 year intervals leads to the increased efficiency of early detection of breast cancer. The present study examined the predictors of repeat mammography. It was hypothesized that higher social support is connected with mammography repeat.

**Methods:**

A cross-sectional study was carried out among 400 women 50 years and older in Sanandaj, Iran. Data was collected by the questionnaire including information on socio demographical variables and measuring social support level. Data was analyzed by SPSS16 software. Multiple logistic regression was used to determine the predictive power of demographic variables and dimensions of social support for repeat mammography.

**Results:**

Women aged 50–55 years had three times odds of repeat mammography compared to women aged 56–60 years) OR, 3.02). Married women had greater odds of repeat mammography compared to single women (*P* < 0.006). The probability of repeat mammography in women with higher social support was 0.93 times greater than the women with lower social support (OR, 0.93; 95 % CI, 0.91–0.95; *P* < 0.0001).

**Conclusions:**

Iranian women are less likely repeat mammography than other Asian women. Identifying the associations between perceived social support and repeat mammography may offer detailed information to allow for future study and guide the development of interventions not only for Iranian women but also for similar cultural that received pay too little attention to date in the breast cancer literature.

## Background

Breast cancer ranks as the first most common cancer among the Iranian women accounting for 21.4 % of various types of cancers [1–3]. The incidence of breast cancer in women ranged from 15 to 84 years old was 22 per 100,000 and the prevalence in this same population was 120 per 100,000 in Iran [4]. In Iranian women breast cancer occurs at least one decade younger than women’s in developed countries [5–7].

Several methods of breast cancer screening including: breast self-examination (BSE), mammography, and clinical breast examination (CBE) [8, 9]. Result of studies indicated that mammography reduced the risk of death from breast cancer among women 40 to 74 years of age amount of 9 % to 32 % [10–13]. In spite of medical recommendations, many women do not receive regular mammograms in Iran [14]. A mammography rate among Iranian women was low (3 % to 12 %) [15–18]. The regular repeat of mammography with 1–2 year intervals leads to the increased efficiency of early diagnosis of breast cancer [19, 20]. Studies conducted in some Europeans countries report the rate of repeat mammography 27–79 % [20–22]. The findings of a study in America showed that the rate of repeat mammography among the women under study was 72.2 % [22]. Also, the results of a study in Iran reported the regular repeat mammography to be 5.7 % [23].

Various definitions have been provided for repeat mammography. Haber defined repeat mammography in their study as having one mammography during the past 12 to 30 months [24]. Rakowski defined as having mammogram within the past two years and 3–11 mammograms within the past 6 years [25]. Further, Taylor defined repeat mammography in their study as having more than one mammography from 50 years onwards [26]. Zabka’s definition of the term was performing one mammography over the two past years [27]. In Rakowski [22] repeat mammography was defined as reporting a most recent exam within the previous 2 years and a next most recent exam within the 2 years before that. Despite the controversies in the provision of one consistent definition of repeat mammography, it has a high diagnostic value if it is repeated regularly [20, 28].

To increase the repeat mammography, the factors influencing this behavior should be identified. It is known that factors such as: fear of lump detection in the breast, anxiety, stress, and worrying about the costs [29, 30] have been as the barriers to repeat mammography. Moreover, factors such as age, perceived susceptibility and severity [23] were associated with repeat mammography. Educational level, income, insurance status, married status, regular visit by the physician, clinician’s advice for mammography, and easy access mammography facilities, family history of breast cancer [22, 31–34] reported as effective factors for mammography. Contribution of social support to mammography behavior is shown [32, 35, 36].

Including social support into women’s health promotion and developing interventions to decrease disease and increase wellness among women are noteworthy. Social support can offer help, directly encouraging preventive behaviors. In addition, it simplifies access to information and transference of knowledge, and delivers encouragement [37, 38]. Social support (emotional, instrumental, informational, and positive social interaction) increase the sense of self-efficacy for overcoming the perceived barriers (emotional, logical, and financial) of mammography [39]. It is documented, increased performance of breast cancer screening behaviors was correlated with high levels of social support [37]. In addition quoted that social support as an influential factor for fostering breast cancer screening behaviors [36, 40, 41]. Social support is a construct which has direct and indirect effects on health, It is a multidimensional concept that is defined aid and assistance exchanged through social relationships and interpersonal transactions or is defined to deal of affection, companionship, care, respect, attention and support received by individual or groups, such as family members, friends and significant others [42, 43]. Social support contained five dimensions (1) emotional support includes the expression of positive affect, e.g., empathy, love, trust, and caring), (2) informational support (the offering of advice, information, guidance or feedback), (3) tangible support (the provision of material aid or behavioral assistance or provision of tangible aid and services that directly assist a person in need), (4) positive social interaction (the availability of other persons to do fun things with you), and (5) affectionate support (involving expressions of love and affection [43, 44].

Determining the correlates of repeat mammography for developing appropriate intervention programs for different groups is important. In the Iranian culture, family relationships, interpersonal relationships, and social networks are influential factors in behavior formation. The findings of a research in Iran emphasized the supportive role of social networks in performing the mammography behavior and mentioned social support as a guideline for performance [45]. No studies have been carried out in Iran on the correlation between social support and repeat mammography. The present study investigated the predictors of repeat mammography. It was hypothesized that higher social support is associated with mammography repeat.

## Methods

A cross-sectional study was carried out among women 50 years and older in Sanandaj, Iran. The inclusion criteria were (1) being 50 years and older, (2) not having a history of breast cancer, (3) not being pregnant or breastfeeding, and (4) having at least one mammogram in the past two years. The list of women aged 50 years and more obtained from the health care centers and they were contacted by phone. In this contact, they were asked whether they had performed mammography at least once over the past two years. Those who answered positively were included in the study and the women with a negative answer excluded. Women with a history of at least one mammography over the past two years and another mammography over the two years before the last mammography were considered as repeat mammography. Women with at least one mammography over the past two years without any other mammography during the two years before the last mammography were considered as non-repeat mammography. The sample size for the study was 400 women of aged 50 years and older. Of these 400 women, 25 cases with incomplete or missing data were deleted, yielding a final sample size of 375.

Data collected by questionnaire in two parts. The first section of the questionnaire was developed to obtain information on socio-demographical variables, having breast cancer screening history (BSE, CBE), history of breast cancer in family, and having breast health problems. The repeat mammography as outcome variable was defined having history of at least one mammography over the past two years and another mammography over the two years before the last mammography.

The second section of the questionnaire was devoted to measuring social support level. The Medical Outcome Study (MOS) instrument was applied [44]. The validity and reliability of the questionnaire was verified by the researchers as the following: The scale was translated using a standard forward-backward translation technique [46]. First, the English version was translated into Farsi independently by 2 professional translators, and a provisional version was provided. Then, 2 bilinguals converted the translated instrument into the original language (English) to monitor retention of the original meaning in the source language. Finally, item-by-item were compared by 1 bilingual, between the back-translated English and the original English versions to make sure the translation was conceptually and linguistically appropriate. Finally, there was no significant difference between original English version and translated version. To determine content validity, a panel of Iranian experts, which included 2 health education professors, 3 gynecologists, a psychologist, and 2 public nursing professors, then reviewed the instrument to determine the cultural appropriateness of the translated tool. The purpose was to ensure that it could be understood by Iranian women and in the most appropriate terms. Translated MOS scale was then reviewed to determine the cultural appropriateness and to validate the translated tool. The panel experts proposed some changes in several items at Farsi version as follows:

Question 2 “Someone to give you information to help you understand a situation” to be changed into: “Someone to give you information to help you understand how to get a mammogram”. Question 3: “Someone to give you good advice about a crisis” to be changed into “Someone to give you good advice about getting mammogram and being on regular”. Question 4 “Someone to confide in or talk to about yourself or your problems” was changed into: “Someone to confide in or talk to about yourself or your questions related to mammogram procedure”. Question 6: “Someone to share your most private worries and fears with” changed to “Someone to share your most private worries and fears regarding with mammogram procedure”. Question 9: “Someone to help you if you were confined to bed” changed into “There is someone to accompany me for physician visiting”. Question 11: “Someone prepares your meals if you were unable to do it yourself” changed into “There is someone to do the cooking for you if mammography procedure that day takes a long time”. Question 12: “Someone to help with daily chores if you were sick” changed into “Someone to help with daily chores if you must spend too much time to get a mammogram”.

Furthermore, the revised translation version of MOS was tested on 20 females (with a history of mammography) to ensure of its clarity and understandability for Iranian women.

To establish the construct validity of the questionnaire, exploratory factor analysis was used. Based on exploratory factor analysis, the questionnaire items were loaded on three factors. The range of factor loading was 0.44–0.72 for the first factor (emotional/informational support), 0.54–0.60 for the second factor (tangible support), and 0.45–0.72 for the third factor (positive social interaction and affectionate support). The reliability coefficient for each scale was calculated using: a) Cronbach’s alpha; b) corrected item-total correlation at least 0.30). The results showed alpha coefficients for the 3 subscale ranging from .72 to .90. Corrected item-total correlation ranged from 0.36–0.75, which means the items are sufficiently related and contributed to score measurement. The Cronbach’s alpha coefficient of the whole questionnaire was 0.91. Ultimately, a 19-item questionnaire with a five-point Likert-type scale was developed including the following three subscales: 1. emotional/informational support including 7 items, 2. the tangible support with 4 items, and 3. positive social interaction and affectionate support with 8 items. For all questions, five answer options were available: “none of the time,” “a little of the time,” “some of the time,” “most of the time,” and “all of the time” [44]. Each participants score was calculated by summing up of total points of all the items. The range of scores was 19–95. A lower score indicated lower social support and a higher score displayed higher social support. The Ethical Committee of Kurdistan University of Medical Sciences approved the study. Prior to participation, investigators sent a written information sheet and consent form for the participants to sign.

Data analyzed by SPSS software16 version. Independent *T*-test and Chi-Square test were used to assess differences in demographic variables (age, marital status, education, employment status, healthcare insurance coverage), breast self-exam(BSE), clinical breast exam (CBE), history of breast cancer in family, and having breast health problems across two groups (women with and without repeat mammogram). To determine the predictive power of demographic variables (age, marital status, education level, employment, and insurance coverage) and dimensions of social support (emotional/informational support, instrumental, affectionate, and positive social interaction) for repeat mammography, multiple logistic regression was used with the repeat mammography group as the reference group.

## Results

Participants had a mean age of 54.82 (SD = 7.42) years (rang = 50–68). Also 27.2 % of participants had a repeat mammography. Details about demographical variables are demonstrated in Table 1. There were no significant differences between women with and without repeat mammogram regarding these variables.

**Table 1:** Distribution of women by socio -demographic characteristics

Characteristics	N (%)
Age	
51–55	150 (40)
56–60	139 (37.1)
> 60	86 (22.9)
Education	
Illiterate	97 (25.9)
Primary and high school	102 (27.2)
Diploma	79 (21.1)
College or above	95 (25.3)
Marital status	
Married	360 (96)
Single	15 (4)
Employment	
Employed	129 (34.4)
Unemployed	242 (64.5)
Health care insurance coverage	
Yes	301 (80.3)
No	71 (18.9)
History of personal breast problem	
Yes	160 (42.7)
No	211 (56.3)
Repeat mammogram	
Yes	102 (27.2)
No	273 (72.8)
Date last mammography	
1 years ago	109 (29.1)
1–2 years ago	100 (26.7)
2–3 years ago	56 (14.9)
> 3 years ago	32 (8.5)
Date penultimate mammography	
1 years ago	69 (18.4)
1–2 years ago	57 (15.2)
2–3 years ago	25 (6.7)
> 3 years ago	29 (7.7)
Family history of breast cancer in the first-degree relative or sister, mother	
Yes	49 (13.1)
No	326 (86.9)

The results of logistic regression analysis for repeat mammography according to demographic variables are presented in Table 2. Women aged 50–55 years had three times odds of repeat mammography compared to women aged 56–60 years) odds ratio [OR], 3.02; 95 % confidence interval [CI], 1.68–5.42; *P* < 0.001). Also, the probability of repeat mammography for the middle-aged women (56–60 years) was 2.24 more that of the women aged > 60 years (OR, 2.24; 95 % CI, 1.26–3.98; *P* < 0.006). The married women had grater odds of repeat mammography compared to single women (OR, 2.24; 95 % CI, 1.26–3.98; *P* < 0.006). Furthermore, the probability of not repeat mammography for illiterate women was 0.65 times smaller compared to the women with primary or high school education (OR, 0.35; 95 % CI, 0.17–0.71; *P* < 0.004). Women holding a diploma had 0.36 times lower odds of repeat mammography compared to women with academic education (OR, 0.64; 95 % CI, 0.30–1.37; *P* < 0.25). Additionally, the probability of repeat mammography for women with a history of breast problems was six times greater than that of the women who did not mention such a history (OR, 6.19; 95 % CI, 3.45–11.11; *P* < 0.001). The odds of repeat mammography for women who had done their last mammography one year ago were three times greater than the women who had performed it two years ago (OR, 3.08; 95 % CI, 1.36–6.97; *P* < 0.007). Additionally, women with a one-year interval before their last mammography had five times odds of repeat mammography compared to those with a two-year interval (OR, 5.19; 95 % CI, 2.43–11.10; *P* < 0.001). Furthermore, the likelihood of repeat mammography for women with a history of regular breast self- examination (BSE) was 0.88 greater that of the women without such a history (OR, 1.88; 95 % CI, 1.16–3.05; *P* < 0.01). In addition, the probability of repeat mammography for women with CBE was five times greater than that of the women without CBE history (OR, 5.35; 95 % CI, 3.19–8.98; *P* < 0.001). Variables such as employment status, insurance coverage, and a positive family history of breast cancer were not predictors of repeat mammography.

**Table 2:** Odds of being in repeat of mammography screening by socio-demographic factors

Characteristics	OR	CI	*P*
Age			
50–55	3.02	1.68–5.42	.001
56–60	2.24	1.26–3.98	.006
> 60			
Marital status			
Married	2.24	1.26–3.98	.006
Single			
Education			
Illiterate	0.35	0.17–0.71	.004
Primary and high school	0.32	0.16–.66	.002
Diploma	0.64	0.30–1.37	.25
College or above			
Employment			
Employed	1.29	0.78–2.11	.31
Unemployed			
Insurance			
Yes	0.53	0.27–1.02	.06
No			
History breast problem			
Yes	6.19	3.45–11.11	.001
No			
Date last mammography			
1 years ago	3.08	1.36–6.97	0.007
2 years ago	1.34	0.63–2.86	.43
3 years ago	0.71	0.32–1.54	.39
4 years ago			
Date Penultimate Mammography			
1 years ago	5.19	2.43–11.10	.0001
2 years ago	3.52	1.76–7.05	.0001
3 years ago	0.97	0.48–1.97	.94
4 years ago			
History of Breast Self- Exam			
Yes	1.88	1.16–3.05	.001
No			
History of Clinical Breast Exam			
Yes	5.35	3.19–8.98	.001
No			
Family history of breast cancer			
Yes	1.53	0.73–3.19	.25
No			

R: *OR* odds ratio, *CI* confidence interval, *P p*-value

Note: Repeat mammography group as the reference group

The findings presented in Table 3 showed that the mean score of social support was 56.6 among the women with repeat mammography and 48.6 among the women without repeat mammography. In other words, the probability of repeat mammography in women with higher social support was 0.93times greater than the women with lower social support (OR, 0.93; 95 % CI, 0.91–0.95; *P* < 0.001). The mean score of emotional/informational support in women with repeat mammography was significantly greater than the women without repeat mammography (25 vs. 21.4). Moreover, the odd of repeat mammography for women with higher emotional/informational support was 0.12 lower than women with lower emotional/informational support (OR, 0.88; 95 % CI, 0.85–0.92; *P* < 0.0001). The difference between means of tangible or instrumental support and emotional support/positive social interaction was greater in women with repeat mammography compared to the women without it. Receiving greater instrumental support increased the odds of repeat mammography by 0.23. Lower emotional support/positive social interaction decreased the probability of repeat mammography by 0.09 (OR, 0.91; 95 % CI, 0.87–0.96; *P* < 0.001) (Table 3).

**Table 3:** Odds of being in repeat of mammography screening by social support factors

Subscale	OR	95 % CI	*P-value*	N	Means
Total social support					
Repeat	0.93	0.91–0.95	.0001	273	56.6
No Repeat				102	48.6
Emotional/informational support					
Repeat	0.88	0.85–0.92	.0001	273	25
No Repeat				102	21.4
Tangible Support					
Repeat	0.77	0.72–0.84	.0001	273	11.7
No Repeat				102	9
Affectionate/Positive Social Interaction Support					
Repeat	0.91	0.87–0.96	.001	273	19.8
No Repeat				102	18

R: *OR* odds ratio, *CI* confidence interval, *P P*-value

Note: Repeat mammography group as the reference group

## Discussion

The findings of this study showed the rate of repeat mammography was 27.2 %. The percentage of repeat mammography varies in different studies. For instance, the rate of repeat mammography was reported 72.2 % in Rakowski’s study, 44.8 % in Gierisch’s study, and 79 % in Mayne’s study [20–22]. Different definitions of repeat mammography may be the reason for varying rates of repeat mammography. Rakowski defined repeat mammography in their study as having two mammography in the time -table based on the one-year intervals. Gierisch defined it as having the second mammography 10 to 14 months following the first mammography. The present study defined repeat mammography as performing one mammography during the past two years and another mammography performed two years before the last mammography.

Age was one of the determining factors in repeat mammography. Younger women aged 50–55 years had more odds of repeat mammography compared to older women aged 55–60 years and more. As shown by other researchers [22, 23, 25, 35]. One reason for this may be due to the normative belief among the women aged over 60 years to spend more time for familial issues than for their personal health issues [23]. This finding helps to develop tailored interventions for older women to obtain repeat mammography [25, 33, 35].

Women with lower education level showed lower probability of repeat mammography. This is consistent with the findings by the other studies [25, 33, 35]. It also demonstrated that low education level serves as an important risk factor of not presenting for performing mammography through reducing access to health care and unawareness of symptoms of breast cancer [47]. It is additionally possible that women with lower level education obtain less information about the importance of repeat mammography via media as magazines, journals, books, and specifically internet.

The findings of this study revealed that insurance coverage had no effect on repeat mammography. This is not consistent with the results of some studies [16, 25, 48], yet, it is consistent with the findings by previous works [18, 23]. The controversy among the findings of these studies may be attributed to the variety in the coverage of insurance in Iran. There are various systems of insurance in Iran including health care insurance, social security insurance, and various supplementary insurances. In this study, we integrated diffident types of insurance due to sample size. The reimbursement of mammography costs varies among the various Iranian insurance systems possibly leading to changes in our results.

Consistent with the results of previous studies, our findings revealed that married women repeat the mammography more frequently [25, 35, 36]. May be the married women receive more support from their spouse and children encouraging them to commit themselves to mammography. This highlights the effective role of spouse and children in supporting women to perform mammography [49]. Family members and the spouse, in particular, as one of the most important components of the social networks, may play a important role in creating positive subjective norms to encourage mammography. Allen has consequently demonstrated the role of urging and encouragement by one of the family members in repeat mammography [48].

Another factor affecting repeat mammography in this study was a positive history of breast problems (pain, abnormal discharge from the breast, and breast abscess, lump, and cyst). The odds of repeat mammography was greater in women with a positive history of these problems being consistent with the results of other studies [17, 48]. Women with problems like pain, abnormal discharge from the breast, abscess, and cyst will be more concerned with the follow up of their health and have a higher probability of turning to the clinicians and doing diagnostic tests such as mammography. The presence of these problems may serve as guidelines or cues to action and drive the patient more strongly towards performing mammography [50].

In line with the findings by Blanchard [51] and Rakowski [22], women with regular intervals of yearly mammography, had a higher probability of repeat mammography. In other words, women with a one-year interval between their recent mammography and their last mammography but one, had a higher probability of repeat mammography compared to women with irregular interval between mammography. It is showed a history of previous mammography was a strong predictor of adhering to guidelines for mammography [39]. One reason for this may be that the past behavior serves as a strong predictor of future behavior [51].

Performing of BSE and CBE predicted repeat mammography. This connection supported by other researches [32, 35]. Performance of BSE and clinical breast examination (CBE) might be considered as predictors of regular mammography behavior in promotion programs of breast cancer screening behavior. The regular performing of BSE and CBE can serves as an reminder factor among women regarding their breast health status, subsequently, leading them to promote repeat mammography.

The findings of our study revealed no association between a family history of breast cancer and repeat mammography. The studies investigating the correlation between family history of breast cancer and screening behavior are contradictory. The former findings indicated the effect of family history on enhancing the mammography behavior [17, 24, 32]. It revealed that women with a positive history of familial breast cancer had lower odds of repeat mammography owing to fearful experiences such as fear of mastectomy, being painful mammography, and fear of irradiation [23, 29, 32].

The mean scores of social support and its related subscales were greater in women with repeat mammography compared to women without it. The results of the study by Messina and Silva entitled “Correlation between Social Support and Repetition of Breast Cancer Screening Behaviors” which used a questionnaire similar to ours were consistent with our findings [35, 37]. Moreover, our findings were consistent with the results of the study that found relationship between social support and mammography performance”, though, of course, they applied different scales to measure social support compared to ours [36]. Social support might contribute to repeat mammography by providing enabling factor [32]. Furthermore, the effects of social support on other variables such as reduced perceived barriers, increased self-efficacy, and increased perceived benefits for mammography behavior are documented [39]. Social support leads to an increased sense of self-efficacy to overcome the perceived barriers of mammography (emotional, logical, and financial) and fosters the perceived benefits of mammography [39]. The probability of the effect of these interactions on repeat mammography behavior should not be overlooked. To make stronger assertions in this regard, future research seems mandatory.

## Limitations of the study

Results of empirical studies have shown that psychosocial factors related to mammography behavior change during different periods of life. There are also methodological attentions when using independent cross-sectional design to compare differences such as mammography rates, the repeat-to-recent ratio, and correlates of recent and repeat utilization across cross-sectional studies. Utilizing the findings of longitudinal studies may lead to the detection of changes in cognitive factors related to breast cancer screening behaviors. Another limitation was lack of comparison between women regarding the number of repetition of mammography. In other words, no comparison was made between women who performed repeat mammography once in the intended interval and those who repeated it twice or more. There is possible that factors affect recent mammography also influences repeat mammography therefore further research is warranted to detect extremely important correlates of recent and repeat mammography utilization. Moreover, the questionnaires were completed through self-report technique for literate women. Therefore, we relied on the dates of mammography reported by women. Yet, they may have not been very correct and we were not free of error of measurement.

## Conclusions

The diagnostic value of mammography in the early diagnosis of breast cancer is dependent on its regular repetition. Regarding the findings of the present study, the elder women should be urged on to repeat mammography. The results of our study demonstrated that social support was an important influential factor in repeat mammography. Hence, more attempts may be made in this regard to enhance social support for women through health care providers and their family members, relatives, and friends. Identification of sources of social support (spouse, family members, relatives and friends, and health care providers) and their effect on mammography behavior demands more future research. It is also mandatory for any society to design and apply some methods for providing various types of social support to enhance breast cancer screening behaviors based on the present status and socio-cultural conditions. Regarding the innovative technologies, use can be made of cell phone short message service (SMS) and internet (E-mail) to provide the social support for repeat mammography (e.g., women can be reminded of the date of repeat mammography via SMS or E-mail). In addition, some attempt should be made to design and implement programs to encourage women to perform regular BSE and CBE. Performance of BSE and CBE will sensitize women on their breast health status, in turn, leading to an increased repeat mammography. Considering the predictive role of past mammography in performing future mammography, encouraging women to repeat mammography once more, will probably have a positive effect on the rate of its repetition.
